# A Novel Electrochemical Aptasensor Based on a New Platform of Samarium Molybdate Flower-like Nanoparticles@Poly(pyrrole) for Non-Invasive Determination of Saliva CORTISOL

**DOI:** 10.3390/bios12090720

**Published:** 2022-09-03

**Authors:** Zahra Rezapoor-Fashtali, Mohammad Reza Ganjali, Farnoush Faridbod

**Affiliations:** 1 Center of Excellence in Electrochemistry, School of Chemistry, College of Science, University of Tehran, Tehran 141556455, Iran; 2 National Institute of Genetic Engineering and Biotechnology (NIGEB), Tehran P.O. Box 14965/161, Iran

**Keywords:** salivary cortisol, aptasensor, samarium molybdate flower-like nanoparticles, poly(pyrrole), reduced graphene oxide

## Abstract

Cortisol, a famous stress biomarker, can be considered a potential predictor of cardiac diseases in humans. The presence of cortisol in saliva has encouraged researchers to design point-of-care devices for cortisol concentration in biological fluids. Here, human salivary cortisol was analyzed through a new non-invasive voltammetric aptasensor. Although cortisol is an electroactive compound, generally, the reduction in the current peak has been considered; however, this does not show a strong signal on a bare electrode surface, especially at low concentration levels. Hence, in this study, cortisol concentration was measured electrochemically and indirectly by monitoring the difference between electrochemical probe signals in the presence and absence of cortisol. A new polymeric nanocomposite of samarium molybdate flower-like nanoparticles decorated in poly(pyrrole) was electro-synthesized on the surface of a glassy carbon electrode. Then, reduced graphene oxide was cast on the surface. Finally, the cortisol aptamer was immobilized covalently on the reduced graphene oxide. This platform was used to increase the oxidation current peak of the ferricyanide solution as a probe as well as its electrocatalyst. The novel designed polymeric has the potential ability for effective immobilization of aptamers on the electrode surface without decreasing their biological activities. Additionally, it can enhance the probe electrochemical signal. The differential pulse voltammetric method (DPV) was applied as the detection technique. By optimizing the effective parameters, a determination range of 5.0 × 10^−14^–1.5 × 10^−11^ mol/L and a limit of detection of 4.5 × 10^−14^ mol/L were obtained. Selectivity of the proposed aptasensor relative to β-estradiol, progesterone and also prednisolone was studied as well. Finally, cortisol in a healthy human saliva sample was successfully analyzed by the proposed biosensors.

## 1. Introduction

Cortisol, the most famous hormone of the corticosteroid family, is secreted by human adrenal glands [1], stimulated by corticotropin (CRH)-adrenocorticotropin (ACTH) [2]. This hormone has a great effect on our body and health. During stress or injury, the secretion of cortisol can reduce pain, inflammation, and swelling and reduce blood flow from the capillaries to the interstitial space. Cortisol concentration levels vary depending on age, gender, daily trend, and season. Its average concentrations in blood serum, urine, sweat, and saliva are about 550.0 nmol/L [3], less than 280.0 nmol/L [4], 137.9 nmol/L [5], and 15.5 nmol/L [6], respectively. A healthy person has normal daily cortisol levels but may have lower or higher levels than normal at certain times of the day. Excessive cortisol leads to Cushing’s syndrome, whereas if it is insufficient, it can lead to Addison’s disease [7]. In addition, many studies have examined the association between plasma cortisol and the risk of cancer [8]. For example, according to a 19-year study of 18932 women, more than 70% of breast cancer patients had high levels of cortisol [9]. Furthermore, increased levels of cortisol in the body can increase the risk of viral and bacterial diseases by weakening the immune system. In the face of the pandemic coronavirus outbreak and according to recent research on 30 patients with COVID-19, cortisol levels in deceased patients were higher than in living ones [10]. Because cortisol is a stress and alert biomarker, impaired secretion can be effective in cardiovascular disease. Cortisol can bind to receptors called mineralocorticoids (MR). Mineralocorticoid receptors are known to regulate blood pressure by modulating the sodium absorbed by the kidneys [11]. Because increased cortisol levels activate the mineralocorticoid receptor in the heart, measuring plasma-induced cortisol due to oxidative stress can be a factor in predicting heart attacks. Researchers found that 48 h before the heart attack, the level of cortisol in the blood of heart patients was higher than normal [12]. Therefore, this stress biomarker can be very important in predicting myocardial infarction.

Nowadays, there are highly accurate and advanced instrumental analysis methods for accurate measurement of cortisol in biological fluids, such as ELISA-based immunoassay techniques that are portable devices, unlike liquid and gas chromatography [13,14]. Cortisol ELISA assay kits are currently the most common commercial kits that can measure cortisol in saliva, urine, serum, and plasma with acceptable sensitivity to the minimum required volume of a real sample [15]. As cortisol concentrations vary depending on environmental and temporal conditions, the design and fabrication point of care and wearable sensors is critical for Cushing’s, Addison’s, and cardiovascular disease. According to [16], researchers are seeking to commercialize a new aptamer-field-effect transistor-based smartwatch for point of care that can determine cortisol in human sweat in real-time. Aptasensors are more cost-effective than the proposed lock-key sandwich structure for antibody and antigen binding [17]. Aptamers are a short sequence of nucleic acids that can behave similarly to antibodies in selectivity to analytes. According to recent research, two different sequences of aptamers have been used to measure cortisol electrochemically [18,19,20,21,22,23,24]. Although cortisol can be electrochemically reduced due to its chemical structure, the reduction region of this hormone interferes with the reduction of water-soluble oxygen [25]. This interference will reduce the reproducibility of cortisol measurements. Because oxygen dissolves in the environment depending on temperature, a slight change in laboratory conditions can lead to errors in measuring cortisol in very small amounts. Therefore, if cortisol is measured directly, it is necessary to blow inert nitrogen gas into a solution to remove oxygen. On the other hand, the study of direct electrochemical reduction of cortisol even after using aptamers achieved a detection limit (LOD) of about 4.9 mmol/L [21], which is higher than other studies that measured cortisol indirectly with the help of a probe.

Therefore, in this study, a non-invasive determination of salivary cortisol is carried out through an indirect electrochemical method. A new biosensor based on an aptamer was investigated to reduce the LOD and also to increase the reproducibility of the analysis. The biosensor signal in this design is the difference of anodic currents of ferricyanide as an electroactive probe in the presence and absence of cortisol in the test solution recorded by differential pulse voltammetry (DPV). One of the most important benefits of such a design is the elimination of the effect of the capacitive current. Anodic and cathodic currents are generally a set of faradic and capacitive currents in electrochemical methods. Faradic currents are proportional to the analyte concentration, while capacitive currents cannot be attributed to the material concentration. Therefore, the presence of capacitive currents can lead to a positive measurement error. If the studied signals are the difference of anodic currents of ferricyanide, capacitive currents are eliminated, and this is one of the most important advantages of using an electroactive probe. Furthermore, an aptamer, which can trap cortisol molecules by forming the third structure, was used to increase the selectivity of the biosensor. This third structure of the selected aptamer is sensitive only to cortisol molecules, which will prevent its interaction with substances similar to cortisol, such as β-estradiol, progesterone and prednisolone. Additionally, the examination of the probe signal difference in real saliva samples helps greatly remove the possible interference of the matrix in the cortisol measurement. In fact, if the signal difference of the probe containing mentioned interferers is investigated in the presence and absence of cortisol, disturbing factors are eliminated.

In this study, reduced graphene oxide (rGO) was employed as a suitable surface for better immobilization of aptamer molecules through covalent binding between the carboxylic group previously functionalized on rGO with the NH_2_-functionalized aptamer sequence. Furthermore, according to a previous study [26], aptamers have a negative charge and prefer to interact with positively charged surfaces of target proteins. However, immobilizing aptamers on positively charged surfaces may result in the complete opening of the aptamer sequences [27]. rGO can not only increase the anodic current of ferricyanide as an electroactive probe due to the reduction of the electro-resistance of the surface but can also prevent entanglement of aptamers with the help of electrostatic repulsion due to its negative charge [28]. As a result, the possibility of forming a three-dimensional structure of an aptamer increases. Briefly, rGO has a significant role to play in the performance of the designed aptasensor. Firstly, it strengthens the signals of ferricyanide, and secondly, it immobilizes the aptamer by creating a covalent bond and helps a better bio-functioning of the aptamer. Excessive accumulation of negative surface charge can lead to creating a linear conformation of an aptamer that is unable to bind to the target molecule [27]. Thus, rGO can increase aptasensors’ stabilization and performance.

Poly(pyrrole), a very biocompatible substance and common material in designing biosensors, was also used. This polymer is a kind of positively charged conductive polymer [29] that can help rGO possessing negative charges immobilized on a glassy carbon electrode surface. In addition, the electro-polymerization of poly(pyrrole) on the surface of a glassy carbon electrode is easily done with the help of cyclic voltammetry. The anodic current of ferricyanide also increased significantly due to the high conductivity of this polymer, which as a result, can increase the sensitivity of the biosensor in measuring cortisol. However, this polymer made ferricyanide oxidation more difficult. Thus, its anodic current shifted slightly towards the positive potentials after electro-polymerization.

To facilitate the oxidation of ferricyanide, samarium molybdate flower-like nanomaterials were used. Samarium is an electroactive lanthanide element with electrons removed from the 6S and 4F layers. During ferricyanide oxidation, a samarium molybdate nanostructure can facilitate probe oxidation by reducing themselves. These flower-like nanomaterials can not only cause ferricyanide electrocatalysts but also increase the effective cross area of the glassy carbon electrode. These nanoparticles were synthesized by the co-precipitation method. Samarium molybdate nanoparticles, depending on the rate of addition of the anion to the cation, can take different shapes and sizes. In the present study, the novel nano flower-like structure was recognized as the best surface modifier. The synthesized samarium molybdite flower-like nanomaterials were then trapped in the polymer fabric during electro-polymerization. Trapping samarium molybdite flower-lik nanoparticles in the poly(pyrrole) can change the structure of the polymer.

The most common type of poly(pyrrole) bonding is cross-linked chains, though the intrinsic properties of poly(pyrrole) are highly dependent on the conditions of electro-polymerization [29]. The type of cross-chain linkages involves sigma-to-sigma bonds of pyrrole aromatics, which can produce a linear polymer chain. However, according to [30], samarium can act as a metal during poly(pyrrole) synthesis and interacts with pyrrole nitrogen. The presence of samarium molybdate flower-like nanoparticles in the pyrrole monomer solution during electro-polymerization leads to the formation of a metal (samarium)-ligand (pyrrole) complex. In fact, the polymer grows not only by sigma bonding but also by π interactions. This novel nano-flower-like structure and poly(pyrrole) (SmM NPs@PP) simultaneously increase the conductivity of the electrode surface and thus increase the anodic current of ferricyanide. In addition, and according to [31], the presence of molybdate in the structure of samarium molybdite nano-flowers provides protection against corrosion of the polymer film by electrochemical methods.

Briefly, in this study, the surface of a GCE was first modified with SmM NPs@PP. Then, the negative charge rGO was placed on the polymeric layer with the help of electrostatic interaction. The aptamer was next connected to rGO by covalent binding. Finally, the designed biosensor was used in the indirect determination of saliva cortisol through differential pulse voltammetric signals of the ferricyanide probe.

## 2. Materials and Methods

### 2.1. Reagents

Samarium(III) chloride hexahydrate (SmCl_3_.6H_2_O), ammonium molybdate tetrahydrate ((NH_4_)_2_MoO_4_.4H_2_O), europium chloride hexahydrate (EuCl_3_.6H_2_O), ammonium paratungstate tetra-hydrate ((NH_4_)_10_(H_2_W_12_O_42_).4H_2_O), pyrrole monomer, sulfuric acid (H_2_SO_4_), reduced graphene oxide (rGO), potassium ferricyanide (K_3_Fe(CN)_6_), sodium chloride (NaCl), sodium dihydrogen phosphate dihydrate (NaH_2_PO_4_.2H_2_O), sodium hydroxide (NaOH), phosphoric acid (H_3_PO_4_), hydrochloric acid (HCl), tris hydrochloride (tris HCl), ethylenediaminetetraacetic acid (EDTA), 1-ethyl-3-(3-dimethyl aminopropyl) carbodiimide (EDC) and N-hydroxy succinimide (NHS) were provided by Merck company. The aptamer was prepared by Pishgam Biotech company in Iran with the following sequence:

5′−NH_2_-(CH_2_)_6_-AGCAGCACAGAGGTCAGATGCAAACCACACCTGAGTGGTTAGCGTATGTCATTTACGGACC-3′.

### 2.2. Instruments

A DropSens μStat 400 potentiostat/galvanostat with Dropview, a software made in Spain, was used for electrochemical studies. To characterize the nano-flowers by field emission scanning electron microscopy (FESEM), a Hitachi S4160 and an EDS EDAX/AMETEK element, made in Japan, a Rigaku UltimaIV X-ray diffractometer (XRD), made in Japan, and a Bruker spectrum Fourier-transform infrared spectrometer (FT-IR), made in the United States of America, were used. In addition, a 2 mm glassy carbon electrode, an Ag/AgCl/KCl (3.0 mol/L) electrode made by Azar Electrode, Iran and a 2 mm mechanical B_2_ pencil lead made in China were used as a working, reference and auxiliary electrode, respectively.

### 2.3. Samarium Molybdate Flower-like Nanoparticles (Sm_2_(MoO_4_)_3_ NPs), Samarium Tungstate (Sm_2_(WO_4_)_3_ NPs) and Europium Molybdate (Eu_2_(MoO_4_)_3_ NPs) Nanostructure Synthesis

Samarium molybdate flower-like nanoparticles (SmM NPs), like the other two nanostructures, samarium tungstate nanoparticles (SmW NPs) and europium molybdate nanoparticles (EuM NPs), were synthesized by the co-precipitation method, one of the most efficient and easy methods for synthesizing nanoparticles by adding an anionic aqueous solution to the cationic aqueous solution that is being stirred [32]. The time of anion to cation addition and also their concentrations have a large effect on the shape and size of sensitized nanoparticles. In this study, anion and cation concentrations of 0.1 mol/L were added together in about 2 h. Subsequently, a centrifuge was used to separate the sediments from disturbing ions. The precipitate was dried in an oven for about two hours at 80 °C.

### 2.4. Poly(pyrrole) Electro-Polymerization

In order to neutralize the negative charge of reduced graphene oxide and prepare the glassy carbon electrode substrate for the stabilized aptamer, poly(pyrrole) was used, synthesized by electro-polymerization. In this method, pyrrole monomers were contacted with the glassy carbon electrode and electro-synthesized by applying potential with the help of cyclic voltammetry (CV). The easy and fast synthesis of polymer on the GCE surface with only one cycle is one of the most advantages of electro-polymerization. In addition, nanomaterials can be used to increase electro-polymerization efficiency during polymer synthesis. In this study, the effect of samarium molybdate flower-like nanoparticles, samarium tungstate, and europium molybdate nanoparticles on the electro-polymerization efficiency as well as the anodic current of ferricyanide was studied. Thus, 25 mg/L of nanostructures (in distilled water) were added to the pyrrole monomer 0.1 mol/L and sulfuric acid 0.25 mol/L in each test. Then, polymeric nanocomposites were synthesized on the GCE surface by applying a cycle in the potential range of −0.2 to +1.0 volts and a potential scanning rate of 0.05 V/s.

### 2.5. Aptamer-Assisted Cortisol Loading

For aptamer loading, EDC-NHS was utilized. EDC can bind to carboxylic functional groups (COO^−^). Therefore, reduced graphene oxide (rGO) was used for this purpose. Initially, rGO can interact with EDC. Then, NHS can be attached to reduced graphene oxide. After the aptamer enters, the NHS-rGO is broken, and the NH_2_-(CH_2_)_6_-aptamer is covalently attached to the reduced graphene oxide instead. In addition, rGO not only aids in the binding of the aptamer but also reduces its resistance by activating the electrode surface and increasing the oxidation signal of ferricyanide. The negative charge of rGO can cause an electrostatic repulsion between the aptamers and prevents sequence entanglement. Therefore, after electro-polymerization, 2 μL of rGO 24 g/L (in distilled water) was dropped on the nano particles@PP/GCE and dried under the IR light.

**Figure 1:**
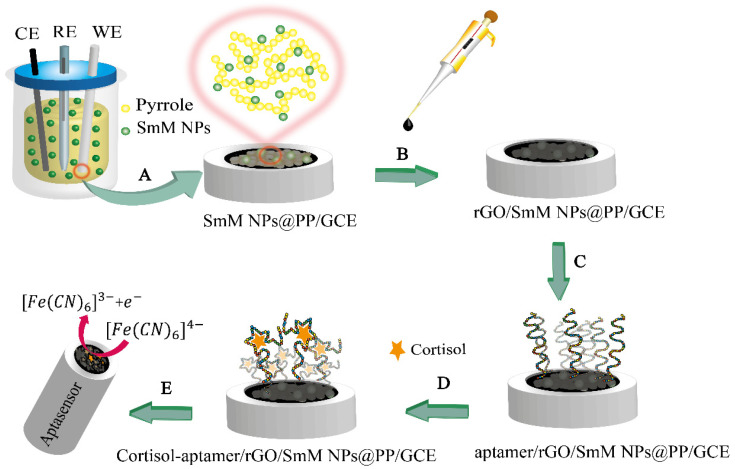
Aptasensor design: (**A**) electro-polymerization to form polymeric nanocomposites; (**B**) rGO dropping; (**C**) aptamer loading; (**D**) aptasensor immersion in the cortisol solution; (**E**) electrochemical investigation of ferricyanide as a probe on the surface of the aptasensor.

The modified electrode was then immersed for 5 min in a mixture of EDC-NHS, 2.0 mmol/L and 5.0 mmol/L, respectively (in 0.01 mol/L phosphate buffer saline (PBS), pH = 7.4) [33]. Subsequently, 2.0 μL of the cortisol aptamer 125.0 mmol/L (in tris HCL-EDTA buffer pH 7.4), which is an optimized incubation concentration of aptamer, was dropped on the modified electrode and dried in the medium (Figure 1). For binding cortisol to the aptasensor surface, the aptamer/rGO/SmM NPs@PP/GCE is immersed in a solution containing the analyte for 15 min, an optimum incubation time.

## 3. Results and Discussions

### 3.1. Investigation of Synthesized Nanostructures on Pyrrole Electro-Polymerization and Ferricyanide Oxidation as a Probe

Nanostructures synthesized during pyrrole electro-polymerization can be trapped in the polymer tissue and increase the effective cross-sectional area of the electrode. Nanoparticles of samarium molybdate, samarium tungstate and europium molybdate were studied to investigate the efficiency of electro-polymerization and the anodic current of ferricyanide as a probe in PBS 0.01 mol/L, pH 7.4 with the help of cyclic voltammetry and differential pulse voltammetry at a potential scanning rate of 0.1 V/s. After electro-polymerization, the anodic current of ferricyanide was 3.56 times that of the bare electrode, while we witnessed an undesirable anodic potential shift of about +0.02 volts relative to the bare electrode. In the presence of samarium molybdate flower-like nanomaterials in the pyrrole monomer solution during electro-polymerization, ferricyanide was oxidized at almost the same bare electrode potential, while the anodic current of ferricyanide increased 4.62 and 1.38 times compared to GCE and poly(pyrrole)/GCE, respectively. Indeed, the presence of samarium in the polymer bed leads to the formation of a metal-ligand complex with nitrogen pyrrole, which transports the polymer bonds from the sigma to the π interaction and causes the polymer to grow in three dimensions other than the cross-linking connection [30]. Without this nanoparticle, poly(pyrrole) has the type of cross-linkage through aromatic rings, while samarium as an interface can be located in the center of the complex and connect other branches of polymer. Thus, this interaction can increase the anodic current of ferricyanide. On the other hand, these nanoparticles can increase the effective cross-area of the electrode, which causes a negative shift of the anodic potential for ferricyanide as a probe. In addition, the molybdite in the structure of the samarium molybdite nano-flower is also very effective in electro-polymerization efficiency. Molybdate can protect the polymer against corrosion during electrochemical reactions in later stages and increase the stability of the electrode. In fact, molybdenum is a key component of “self-healing” poly (pyrrole) films [31].

In order to investigate the effect of other nanoparticles on the electro-polymerization efficiency, europium molybdate nanoparticles were used. Europium has a structure almost similar to samarium, but like samarium, it cannot form a metal-ligand complex with pyrrole. According to [34], the integration of Eu_2_O_3_ into the pyrrole matrix can increase porosity and charge transfer. However, in the present study, europium molybdate nanoparticles could not provide a favorable response. The anodic current of ferricyanide in the presence of europium molybdate increased 0.97 and 3.25 times relative to the bare and poly(pyrrole)/GCE, respectively. In addition, adverse displacement of the anodic ferricyanide potential of 0.08 and 0.10 relative to the bare and poly(pyrrole)/GCE were also observed, respectively. Therefore, the mere presence of molybdate in the structure of nanoparticles is not enough to improve the results. In the following and in order to investigate the effect of samarium, samarium tungstate nanoparticles were used. Tungstate has a structure almost similar to molybdenum, but like molybdate cannot withstand electrochemical corrosion. According to [35], the presence of tungstate as impurities in poly(pyrrole) prevents the polymer defect and, to some extent, increases the corrosion resistance. However, after adding samarium tungstate nanoparticles to the pyrrole solution during electro-polymerization, the results were not as favorable as for SmM NPs@PP/GCE. The anodic current of ferricyanide on the SmM NPs@PP/GCE surface was 3.80 and 1.13 times relative to the bare and poly(pyrrole)/GCE, respectively. Samarium tungstate nanoparticles could not cause ferricyanide electrocatalysts. The positive displacement of the anodic potential of ferricyanide after the addition of samarium tungstate nanostructures relative to the bare and poly(pyrrole)/GCE were 0.03 and 0.01 V, respectively. Therefore, nano flower-like samarium molybdate has the best efficiency in the electro-catalyzing of ferricyanide as a probe and electro-polymerization efficiency.

### 3.2. Samarium Molybdate Follower-like Nanoparticles Characterization

**Figure 2:**
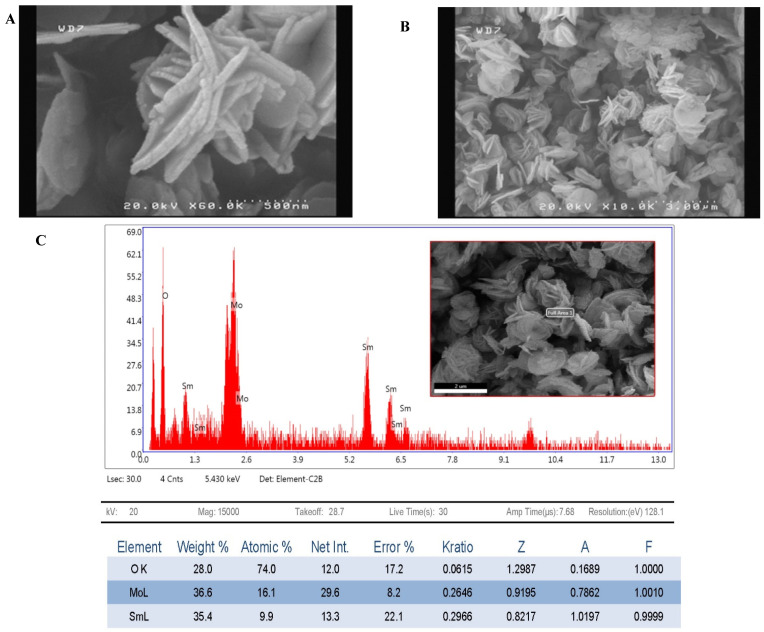

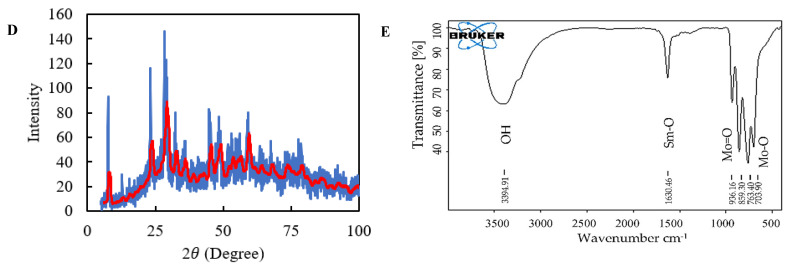
SmM NPs characterization: (**A**) FESEM of SmM NPs with a magnification of 60,000 times; (**B**) FESEM of SmM NPs with a magnification of 10,000 times; (**C**) EDX of SmM NPs; (**D**) XRD pattern of SmM NPs; (**E**) FR-IR spectrum of SmM NPs.

Samarium molybdate flower-like nanoparticles were characterized by FESEM. As shown in Figure 2A,B, samarium molybdate nanoparticles have flower-like structures. The shape and size of molybdate nanoparticles depend on the addition rate of anions to cations [36]. If molybdite anions were added to samarium cations quickly, materials agglomerate and leave the nanoscale, while if it takes about 2 h, nanoscale flower-like structures will form. According to experiments, samarium molybdate flower-like nanoparticles amplify the ferricyanide signal well. Thus, these structures prove that the addition of ions took place at the optimal time. Furthermore, the energy dispersive X-ray analysis (EDX) of samarium molybdate flower-like nanoparticles reveals the constituent elements of these nanoparticles, including samarium, molybdate, and oxygen. These nanoparticles have a monoclinic structure and an XRD pattern with ICDD 24-0997 [37] (Figure 2D). In addition, FT-IR spectroscopy was used to examine functional groups for samarium molybdate flower-like nanomaterials. According to [38,39], molybdenum in the FT-IR spectrum has a wild and complex band of about λ = 600 cm^−1^ that corresponds to Mo-O vibration. Mo=O also gives vibration peaks at wavelengths of approximately 900 cm^−1^ to 1000 cm^−1^. Moreover, the broadband is about 1033 cm^−1^, corresponding to Sm-O [40]. Therefore, according to the IR spectrum (Figure 2E), 703, 763, 859, and 936 cm^−1^ are related to the tensile vibrations of Mo-O and Mo=O joints. Additionally, 1630 cm^−1^ and 3394 cm^−1^ can be related to tensile vibrations of Sm-O and OH, respectively.

### 3.3. Investigation of Electrochemical Performance of Samarium Molybdate Follower-like Nanoparticles, Poly(pyrrole) and rGO on the Ferricyanide’s Anodic Current

We found that all the modifiers used in this study could affect the anodic current of ferricyanide individually and in pairs. Additionally, their mixture together can increase the intensity of ferricyanide anodic current significantly. Initially, the anodic currents of potassium ferricyanide 0.01 mol/L, PBS 0.01 mol/L, and pH = 7.4 were measured before and after a modifier relative to the bare electrode with cyclic voltammetry at a scanning rate of 0.1 V/s. The anodic current of ferricyanide after electrode surface modification with samarium molybdate nanoparticles, polymer and rGO increases by an average of 1.5 times, which represents the effect of each modifier on the signal (Figure S1A–C, Supplementary Data). Then, a simultaneous examination of two modifiers on the electrode surface was studied. When two modifiers were placed on the electrode surface, an average of a 2.5–3.4-fold increase in anodic current was seen compared to a single modifier (Figure S1D–F, Supplementary Data). This supplementary figure shows the role of each of the modifiers in the performance of the designed biosensor. In the absence of any of them, the response of the aptasensor will decrease, and in the next steps, the stability of the biosensor will reduce significantly by weakening the interaction of the aptamer with the electrode surface.

### 3.4. Incubation Study of EDC/NHS Time, Aptamer Concentration and Cortisol Immersion Time

**Figure 3:**
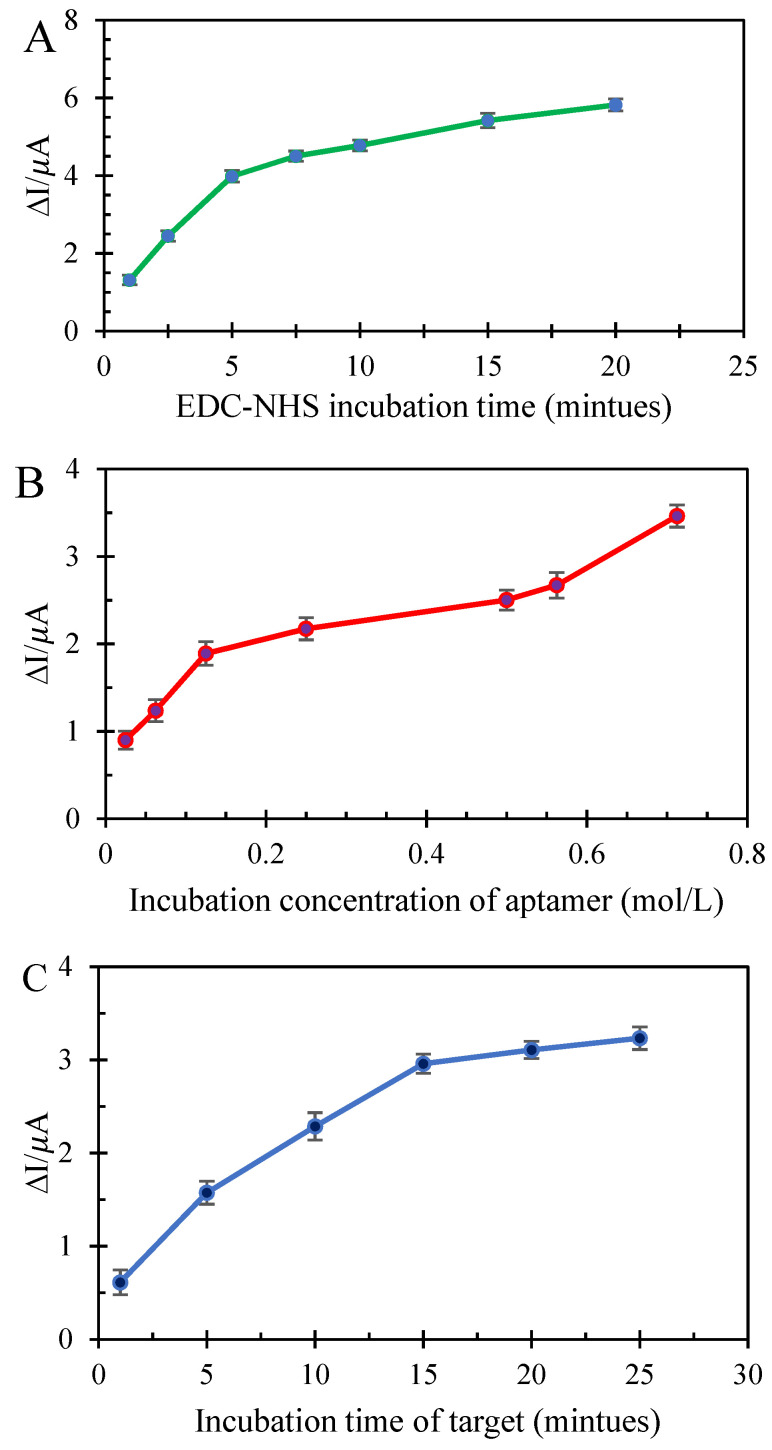
Incubation study of: (**A**) EDC-NHS time; (**B**) aptamer concertation (mol/L); (**C**) aptasensor immersion time in the cortisol solution.

EDC-NHS was used to stabilize the aptamer on the rGO/SmM NPs@PP/GCE surface. For this purpose, the modified electrode was immersed in EDC-NHS solution for 5 min, and then the anodic current of ferricyanide was studied as a probe. The carboxylic acid functional groups of reduced graphene oxide are involved in covalent bonds with NHS, and the electrode surface is slightly passive. Therefore, after immersion, the anodic ferricyanide current decreases. The incubation time of electrode immersion in NHS-EDC was studied. If the electrode was placed in EDC-NHS for more than 5 min, its surface was severely inactivated. However, if the immersion was less than the optimum incubation time, the aptamer cannot be covalently bonded to the electrode surface in later stages. The difference in anodic currents of ferricyanide before and after immersion was plotted relative to the EDC-NHS incubation times. As can be seen in Figure 3A, the rGO-NHS bonding at 1 and 2.5 min was not as good as at 5 min. Additionally, at 7.5–20 min, there is not much difference between the currents of the ferricyanide, which means that the best incubation time is 5 min. In addition, after aptamer stabilization and also trapping cortisol with aptamer, anodic currents decline. Thus, if the ferricyanide current decreases too much at this stage, it can reduce the sensitivity of the method in later stages.

After the aptamer enters, NHS leaves rGO, and the NH_2_-aptamer binds to the reduced graphene oxide. Since the concentration of aptamer is very influential on the results, and due to the negative charge of rGO as well as the aptamer itself, the incubation concentration of the aptamer was also optimized. If the aptamer concentration is too high, it will inactivate the electrode surface, whereas if there is an attempt to determine cortisol at a low aptamer concentration, it cannot interact with the analyte. According to Figure 3B, which shows the difference between anodic ferricyanide currents in the presence and absence of aptamer relative to its concentration, the aptamer concentration of 0.025 mol/L and 0.0625 mol/L could not reduce the anodic ferricyanide currents as well as 0.0125 mol/L. As can be seen in Figure 3B, the slope of the graph is smaller in the range of concentrations of 0.25–0.71 mol/L compared to the 0.02–0.125 mol/L range. Therefore, in this study, 0.125 mol/L was recognized as the optimal aptamer incubation concentration.

Next, the incubation time of the immersion of the aptasensor in cortisol solution was studied. For this purpose, the electrode was immersed in cortisol 7 × 10^−13^ mol/L for 1 to 25 min, and then the anodic currents of ferricyanide were examined according to the immersion time. If the aptamer/rGO/SmM NPs@PP/GCE is immersed in the cortisol solution for less than 15 min, cortisol will not have a chance to be captured by the aptamer, and reciprocally, if the immersion time increases to more than 15 min, there will be no change in the peak intensity (Figure 3C).

### 3.5. PH Studies for the Aptamer/rGO/SmM NPs@PP/GCE

Cortisol does not dissolve at pH below 6 or above 9. Aptamer nucleic acid chains are also broken in highly acidic and alkaline environments. Therefore, pH optimization was performed in an almost neutral medium with the help of cyclic voltammetry at a scanning rate of 0.1 V/s in 0.01 mol/L ferricyanide, PBS 0.01 mol/L. Finally, pH = 7.4 had the best results on the anodic current of the ferricyanide solution (Figure S2, Supplementary Data).

### 3.6. Scanning Rate Studies for the Aptamer/rGO/SmM NPs@PP/GCE

To optimize the potential scanning rate, the anodic current of 0.01 mol/L ferricyanide solution, PBS 0.01 mol/L at pH 7.4 was examined with the help of cyclic voltammetry. For this purpose, the scanning rate was changed from 0.20 to 0.02 V/s. At a high scanning rate, there are a lot of capacitive currents. However, at a very low scanning rate, the opportunity for the electrochemical reaction is limited, and the anodic faradic current decreases. Therefore, the potential scanning rate of 0.1 V/s was introduced as optimal. For comparison and studying the effective surface of the electrode, the Randles–Sevcik equation was used (Equation (1)). According to this equation, the current is related to the potential scanning rate (*v*). Therefore, if the current diagram is plotted in *v*^1/2^ (squared potential scanning rate), the effective area of the electrode can be calculated from the slope of this diagram. An electrochemical study was performed for ferricyanide. Since the concentration (C), the number of electrons exchanged (n), and the diffusion coefficient (D) of this material are constant, the ratio of the slopes is proportional to the effective cross-section of the electrode. Therefore, by changing the potential scan rate of the GCE and the aptamer/rGO/SmM NPs@PP/GCE in ferricyanide 0.01 mol/L, PBS 0.01 mol/L, pH 7.4, the cross-sectional area of the modified electrode was 5.15 times that of the bare one. This increase in the cross-section of the electrode indicates the effect of the modifiers on the ferricyanide anodic current (Figure S3, Supplementary Data).

I = 2.69 × 10^5^ × n^3/2^ × A × D^1/2^ × C × *v*^1/2^ (1)

### 3.7. Electrochemical Impedance Spectroscopy Studies to Qualitatively Evaluate the Aptasensor Surface

**Figure 4:**
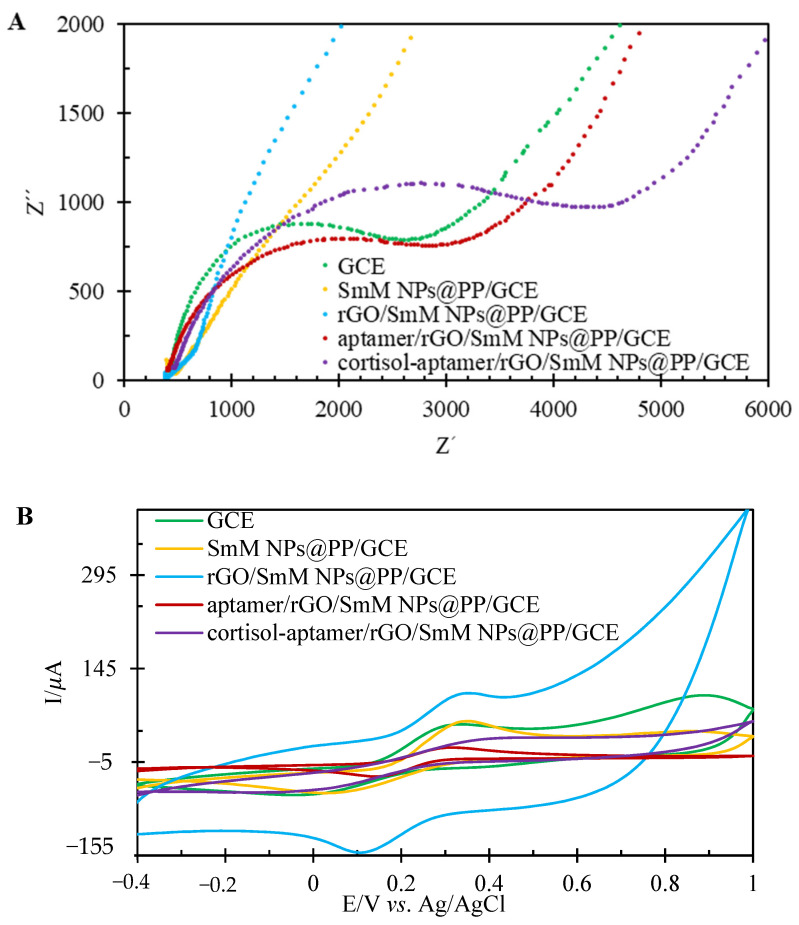
Investigation of electrode surface after each surface modification step comparison in ferricyanide 0.01 mol/L, PBS 0.01 mol/L, pH 7.4 with the help of; (**A**) EIS; (**B**) cyclic voltammetry vs. Ag/AgCl/KCl (3 mol/L).

For the surface resistance study, electrochemical impedance spectroscopy (EIS) was used. In EIS, the diameter of the semicircle indicates the surface resistance. As shown in Figure 4A, the diameter of the semicircle decreased after the surface modification with the help of polymeric nanocomposites (SmM NPs@PP) compared to the bare electrode. This change indicates an increase in the effective electrode surface. In addition, after rGO dropping, the semicircle diameter almost disappears, which indicates an increase in the conductivity of the electrode surface. In the following, the surface resistance increased after aptamer loading. When cortisol was trapped by the aptamer, the diameter of the semicircle became extremely large, indicating that the surface became passive.

The result obtained through EIS corresponds exactly to cyclic voltammetry. As shown in Figure 4B, after the GCE modification with the polymeric nanocomposite (SmM NPs@PP), the anodic ferricyanide current increases dramatically. Additionally, the anodic ferricyanide current also increased, as expected, after rGO dropped. Subsequently, binding of the aptamer to the surface of the modified electrode reduces the conductivity. Therefore, we saw a decrease in the intensity of the ferricyanide anodic current due to the growth of surface passivity. After the aptamer/rGO/SmM NPs@PP/GCE immersion into the cortisol solution, the anodic current of ferricyanide was again reduced.

### 3.8. Reproducibility, Repeatability and Stability Studies for the Aptamer/rGO/SmM NPs@PP/GCE

To validate the proposed method, the repeatability and stability of the aptasensor were investigated. Relative standard deviation (RSD) was used to study the repeatability and reproducibility of the proposed aptasensor. To calculate these parameters, the anodic current of ferricyanide (0.01 mol/L in PBS 0.01 mol/L, pH 7.4) on the aptamer/rGO/SmM NPs@PP/GCE was recorded 5 times with differential pulse voltammetry at the potential scan rate of 0.1 V/s. The relative standard deviation was calculated at 1.32%, which indicates the repeatability of this method. Reproducibility of the aptasensor was tested in the same conditions by three similar prepared electrodes; the RSD was found to be less than 3.7%. In addition, to study the stability of the biosensor, the performance of the proposed aptasensor was studied within 1 month. A gradual decrease of 2.15% was seen after 7 days. During this time, the aptasensor was kept at a temperature of 5 to 7 °C with the help of a sterilized cover on the electrode. 

### 3.9. Evaluation of Cortisol Interaction on the Aptamer/rGO/SmM NPs@PP/GCE Surface and Its Specific Response to β-Estradiol, Progesterone and also Prednisolone

Differential pulse voltammetry (DPV) was used to quantitatively investigate the relationship between the ferricyanide anodic current and the cortisol concentration. After cortisol trapping by the aptamer, the electrode was placed in a ferricyanide solution. Then, the anodic current intensity of ferricyanide was studied. The difference between the anodic current of ferricyanide 0.01 mol/L, PBS 0.01 mol/L, pH = 7.4 in the presence and absence of cortisol can be a good sensing signal for the calibration curve.

**Figure 5:**
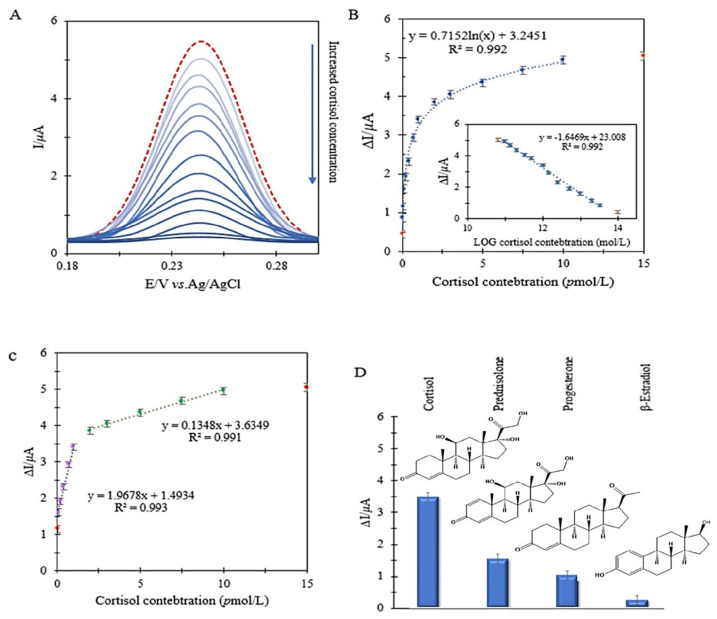
Electrochemical investigation of aptasensor in ferricyanide 0.01 mol/L, PBS 0.01 mol/L, pH = 7.4 with the help of DPV vs. Ag/AgCl/KCl (3 mol/L at the potential scanning rate of 0.1 V/s): (**A**) voltammogram of changes in anodic current of probe relative to cortisol concentration; (**B**) logarithmic correlation of ferricyanide anodic current changes with cortisol concentration; (**C**) two linear areas of the calibration curve of the anodic current changes of the probe in terms of cortisol concentration; (**D**) evaluation of selectivity of designed aptasensor compared to other similar compounds in concentrations equal to 1 *p*mol/L of β-estradiol, progesterone, and prednisolone.

In this way, cortisol concentrations between 5 × 10^−14^ and 1.5 × 10^−11^ mol/L were examined. As shown in Figure 5B, in a wide linear range of cortisol concentrations, there is a logarithmic relationship between cortisol concentrations and sensing signals which shows the applicability of the aptasensor in a rather wide concentration range. Although such a wide applicable linear range is obtained by applying the log of concentration, the sensitivity in the whole range of concentration is not the same. At low concentrations of cortisol, the active binding sites on the surface are easily available for the analyte, while increasing the concentration causes diffusion to the surface of the biosensor, and accessibility to the active sites becomes difficult. This causes two linear measurement regions with different sensitivities, 1.0 × 10^−13^–1.0 × 10^−12^ mol/L and 1.0 × 10^−12^–1.0 × 10^−11^ mol/L, with R^2^ = 0.993 and 0.991, respectively (Figure 5C). These two areas have two different slopes (including 1.97 µAL/pmol and 0.14 µAL/pmol, respectively) and, in fact, sensitivity. In addition, the lowest concentration of the analyte that makes a significant current difference compared to the probe is 4.5 × 10^−14^ mol/L (LOD). It should be noted that, in the design of biosensors, the goal is to reduce the detection limit of the measurement in order to minimize the sampling volume of biological fluids and use other fluids like saliva instead of plasma or blood. Thus, the first linear part is to be used for real sample analyses.

Then, in order to evaluate the aptasensor’s specificity, β-estradiol, progesterone and also prednisolone, which have similar structures to cortisol, were used. As shown in Figure 5D, the difference in anodic ferricyanide currents after incubation with β-estradiol at the exact incubation time optimized for cortisol, i.e., 15 min, demonstrated slight changes compared to the blank form. However, progesterone and prednisolone responded better than β-estradiol due to their structural similarity to cortisol. In common clinical measurements of cortisol, banning the use of prednisolone tablets or ampoules about 24 h before sampling is necessary because prednisolone, due to its structure being very similar to cortisol, can cause errors in test results. For this reason, as in previous research, the behavior of the biosensor in the mixture of cortisol and interferences should be investigated. Nevertheless, in this study, the designed aptasensor was able to eliminate interference effects without simultaneously examining them with cortisol with the help of the signal difference investigation. In addition, this approach caused an acceptable selectivity towards cortisol. In fact, in order to study the real samples, not only do signal difference investigations remove capacitive currents, but they can also minimize interfering errors.

As shown in Table 1, two types of aptasensors measured cortisol directly. Expensive materials such as gold electrodes, DNA-based superlattices (DNA SL), or magnetic nanoparticles (MNP) have been used in these studies, whereas the detection limit of these two aptasensors was about 10 and 130 *p*mol/L. In this study, we were able to minimize the effect of capacitive currents as well as systematic errors by using ferricyanide as a probe and achieved a limit of detection of 0.05 *p*mol/L. This detection limit is less than the LOD that electrochemical researchers have achieved in recent years for cortisol. This low detection limit helped us to require fewer real samples.

### 3.10. Real Saliva Samples Analysis on the Aptamer/rGO/SmM NPs@PP/GCE

Finally, in order to evaluate the use of the aptasensor, we tested it with the saliva of a healthy human. Since the level of cortisol concentration reaches its maximum in the 2.0 h after waking up, the early morning hours were selected for sampling. The mouth was rinsed with cold water and a toothbrush before sampling to prevent food particles from entering the sample. The sampling container was a 250.0 mL syringe. Sterile cotton as a filter was packed on the syringe. About 1.0 mL of saliva was collected for multiple experiments. After passing the saliva through the cotton filter, it was stored in a sterile sample container at 5.0 °C. Since each test requires just 12.5 μL of saliva, it must be diluted 2000.0 times with PBS 0.01 mol/L, pH 7.4. After preparing the saliva sample, it was studied with the help of the standard addition method with 5 replications.

In this method, a constant amount of the unknown sample is added to solutions of the variable standard amount of cortisol. There was 0.01 mol/L ferricyanide as a probe inside each of the solutions. Then the anodic currents of ferricyanide were studied by the differential pulse voltammetry method vs. Ag/AgCl/KCl (3 mol/L) at a potential scanning rate of 0.1 V/s. Since the designed aptasensor has two linear regions with two different slopes, the region that requires the least amount of real saliva sample was selected. Because the linear slope of the selected area (1.97 µAL/*p*mol) is higher than the second area (0.14 µAL/*p*mol), it increases the sensitivity of the method at low cortisol concentrations. In addition, by increasing the degree of dilution of the complex matrix of the real sample, the interference of other materials in the measurement was reduced.

The signal was the difference in the anodic current of ferricyanide, which has interfering substances including β-estradiol, progesterone, and prednisolone with a concentration of 1 *p*mol/L, in the presence and absence of cortisol. In fact, firstly, the ferricyanide signal was recorded in the vicinity of interfering substances (**I_interference_**), and then the ferricyanide anodic current was measured in the solutions containing the real sample (**I_interference+cortisol_**). In this way, the effect of the intervenors is eliminated, and the ferricyanide signal difference (**I_interference_**_+**cortisol**_ − **I_interference_** = **I_cortisol_**) will only be proportional to the cortisol concentration. The concentration of cortisol in saliva was obtained as 4.3 × 10^−9^ mol/L with RSD = 4.8%. Since the amount of healthy human saliva should be in the range of 3.0 × 10^−9^ mol/L to 1.5 × 10^−8^ mol/L, the levels of cortisol in this individual were normal.

## 4. Conclusions

This study aimed to design and fabricate an aptasensor based on the indirect electrochemical method to measure cortisol, which is a biomarker of stress and one of the most important hormones. Because the concentration of cortisol in the body varies at different times of the day, its continuous monitoring with the help of point of care sensors is very important for patients with depression, Cushing’s and Addison’s, as well as cardiovascular diseases. In this research, cortisol was studied indirectly by voltammetric methods with the help of ferricyanide as a probe. Since the difference in anodic currents of ferricyanide was studied before and after the introduction of cortisol, it can eliminate the effect of capacitive currents as well as systematic errors from the method, which increased the reproducibility. The aptasensor designed in this study has been modified with the help of novel nano polymeric (SmM NPs@PP), which can reduce the negative charge of reduce graphene oxide. Samarium molybdate flower-like nanomaterials@poly(pyrrole) can not only increase the bond stability of the aptamer but also increase the conductivity, the effective cross area of the electrode and also create the electrocatalytic property of the ferricyanide. Subsequently, the aptamer passives the surface of the electrode when cortisol enters. After optimization of pH, the scanning rate, incubation time and concentration, the anodic current of ferricyanide 0.01 mol/L, PBS 0.01 mol/L, pH = 7.4 was studied by DPV vs. Ag/AgCl/KCl (3 mol/L). This biosensor showed high repeatability, stability and selectivity with a determination range of 5.0 × 10^−14^–1.5×10^−11^ mol/L, with two linear ranges: 1.0 × 10^−13^–1.0 × 10^−12^ mol/L and 2.0 × 10^−12^–1.0 × 10^−11^ mol/L, as well as a detection limit of 4.5 × 10^−14^ mol/L. Finally, the efficiency of the aptasensor in a real saliva sample two hours after waking up was evaluated successfully.

## Figures and Tables

**Table 1:** Comparison table of aptamer-based electrochemical sensors for measuring cortisol in biological fluids by using electrochemical methods during the last 5 years. (* The lowest detection limits.).

Modified Electrode	Voltammetric Method	LOD (*p*mol)	Determination Range (*p*mol)	Real Sample (Human)	References
Multi-walled carbon nanotube-copper porphyrin-magnetic nanoparticles-aptamer/screen printed electrode	DPV (direct determination)	10.0	1 × 10^2^–5.0 × 10^4^	Saliva	[19]
Aptamer/polyimide substrate patch with sputtered Zn-O	EIS	2.7 × 10^3^	2.7 × 10^3^–7.1 × 10^5^	Sweat	[20]
Polydimethylsiloxane@cellulose nanocrystal/carbon nanotube@aptamer	CV	5.0 × 10^3^	6.9 × 10^3^–9.7 × 10^4^	Sweat	[21]
Aptamer/streptavidin magnetic beads/screen-printed graphene electrodes	EIS	5.8	2.7×10^2^–2.7×10^5^	Sweat	[22]
BSA/aptamer/Ni(OH)_2_@N-CN-Tube/GCE	CV	3.0 × 10^−1^	1.0–8 × 10 and 1.0 × 10^2^–2.5 × 10^4^	Saliva and serum	[23]
Aptamer/DNA SL/gold electrode	EIS (Direct determination)	1.3 × 10^2^	5.0 × 10^2^–1.0 × 10^4^	Saliva	[24]
Aptamer/rGO/SmM NPs@PP/GCE	DPV	4.5 × 10^−2^ *	5.0 × 10^−2^–1.5 × 10	Saliva	This study

## Supplementary Materials

The following supporting information can be downloaded at: https://www.mdpi.com/article/10.3390/bios12090720/s1, Figure S1: Comparison of electrode surface with the help of cyclic voltammetry vs. Ag/AgCl/KCl (3 mol/L) at potential scanning rate 0.1 V/s in ferricyanide 0.01 mol/L, PBS 0.01 mol/L, pH 7.4: (A) the PP/GCE and the bare electrode; (B) the SmM NPs/GCE and the bare electrode; (C) the rGO/GCE and the bare electrode; (D) the PP/GCE, SmM NPs /GCE with the SmM NPs@PP/GCE; (E) the PP/GCE, rGO/GCE with the rGO/PP/GCE; (F) the SmM NPs/GCE, rGO/GCE with the rGO/SmM NPs/ GCE; Figure S2: pH studies for the aptamer/rGO/SmM NPs@PP/GCE with the help of cyclic voltammetry vs. Ag/AgCl/KCl (3 mol/L) at potential scan rate 0.1 V/s in ferricyanide 0.01 mol/L, PBS 0.01 mol/L; Figure S3: Investigation of the potential scanning rate on anodic currents of ferricyanide 0.01 mol/L, PBS 0.01 mol/L, pH 7.4 with the help of cyclic voltammetry vs. Ag/AgCl/KCl (3 mol/L) and the Randles-Sevcik equation to calculate the effective cross-section of (A) the bare GCE electrode; (B) the aptamer/rGO/SmM NPs@PP/GCE.

## References

[1] Taves M.D., Gomez-Sanchez C.E., Soma K.K. (2011). Extra-adrenal glucocorticoids and mineralocorticoids: Evidence for local synthesis, regulation, and function. Am. J. Physiol. Metab..

[2] Yoshio Takei H.A., Kazuyoshi T. (2015). Handbook of Hormones, Comparative Endocrinology for Basic and Clinical Research.

[3] Lee D.Y., Kim E., Choi M.H. (2015). Technical and clinical aspects of cortisol as a biochemical marker of chronic stress. BMB Rep..

[4] Butts C.D., Bloom M.S., Frye C.A., Walf A.A., Parsons P.J., Steuerwald A.J., Ilonze C., Fujimoto V.Y. (2014). Urine cortisol concentration as a biomarker of stress is unrelated to IVF outcomes in women and men. J. Assist. Reprod. Genet..

[5] Tu E., Pearlmutter P., Tiangco M., Derose G., Begdache L., Koh A. (2020). Comparison of Colorimetric Analyses to Determine Cortisol in Human Sweat. ACS Omega.

[6] Pearlmutter P., DeRose G., Samson C., Linehan N., Cen Y., Begdache L., Won D., Koh A. (2020). Sweat and saliva cortisol response to stress and nutrition factors. Sci. Rep..

[7] Brooke A.M., Monson J.P. (2017). Addison’s disease. Medicine.

[8] Larsson S.C., Lee W.-H., Kar S., Burgess S., Allara E. (2021). Assessing the role of cortisol in cancer: A wide-ranged Mendelian randomisation study. Br. J. Cancer.

[9] Al Sorkhy M., Alfahl Z., Ritchie J. (2018). Cortisol and Breast Cancer: A review of clinical and molecular evidence. Ann. Cancer Res. Ther..

[10] Ramezani M., Simani L., Karimialavijeh E., Rezaei O., Hajiesmaeili M., Pakdaman H. (2020). The Role of Anxiety and Cortisol in Outcomes of Patients with Covid-19. Basic Clin. Neurosci. J..

[11] Sandle G.I., Keir M.J., Record C.O. (1981). The Effect of Hydrocortisone on the Transport of Water, Sodium, and Glucose in the Jejunum. Scand. J. Gastroenterol..

[12] Yamaji M., Tsutamoto T., Kawahara C., Nishiyama K., Yamamoto T., Fujii M., Horie M. (2009). Serum Cortisol as a Useful Predictor of Cardiac Events in Patients with Chronic Heart Failure: The impact of oxidative stress. Circ. Heart Fail..

[13] Heckmann M., D’Uscio C.H., Steckel H., Neuhaeuser C., Bödeker R.-H., Thul J., Schranz D., Frey B.M. (2014). Reduction in Cortisol Inactivation is Part of the Adrenal Stress Response to Cardiac and Noncardiac Pediatric Surgery: A Prospective Study Using Gas Chromatography-Mass Spectrometry Analysis. Horm. Metab. Res..

[14] Li C., Zhang Z., Liu X., Shen K., Gu P., Kang X. (2017). Simultaneous quantification of cortisol and cortisone in urines from infants with packed-fiber solid-phase extraction coupled to HPLC–MS/MS. J. Chromatogr. B.

[15] Somrudee Saiyudthonga P.S., Trongwongsab T., Srisurapanon S. (2010). Comparison between ECL and ELISA for the detection of salivary Cortisol and determination of the relationship between Cortisol in saliva and serum measured by ECL. ScienceAsia.

[16] Wang B., Zhao C., Wang Z., Yang K.A., Cheng X., Liu W., Yu W., Lin S., Zhao Y., Cheung K.M. (2022). Wearable aptamer-field-effect transistor sensing system for noninvasive cortisol monitoring. Sci. Adv..

[17] Sun K., Ramgir N., Bhansali S. (2008). An immunoelectrochemical sensor for salivary cortisol measurement. Sens. Actuators B Chem..

[18] Sanghavi B.J., Moore J.A., Chávez J.L., Hagen J.A., Kelley-Loughnane N., Chou C.-F., Swami N.S. (2016). Aptamer-functionalized nanoparticles for surface immobilization-free electrochemical detection of cortisol in a microfluidic device. Biosens. Bioelectron..

[19] Fernandez R.E., Umasankar Y., Manickam P., Nickel J.C., Iwasaki L.R., Kawamoto B.K., Todoki K.C., Scott J.M., Bhansali S. (2017). Disposable aptamer-sensor aided by magnetic nanoparticle enrichment for detection of salivary cortisol variations in obstructive sleep apnea patients. Sci. Rep..

[20] Pali M., Jagannath B., Lin K.-C., Upasham S., Muthukumar S., Prasad S. (2021). CATCH (Cortisol Apta WATCH): ‘Bio-mimic alarm’ to track Anxiety, Stress, Immunity in human eccrine sweat. Electrochim. Acta.

[21] Mugo S.M., Alberkant J., Bernstein N., Zenkina O.V. (2021). Flexible electrochemical aptasensor for cortisol detection in human sweat. Anal. Methods.

[22] Pusomjit P., Teengam P., Thepsuparungsikul N., Sanongkiet S., Chailapakul O. (2021). Impedimetric determination of cortisol using screen-printed electrode with aptamer-modified magnetic beads. Microchim. Acta.

[23] Roushani M., Hosseini H., Hajinia Z., Rahmati Z. (2021). Rationally designed of hollow nitrogen doped carbon nanotubes double shelled with hierarchical nickel hydroxide nanosheet as a high performance surface substrate for cortisol aptasensing. Electrochim. Acta.

[24] Cantelli L., Paschoalino W.J., Kogikosky S., Pessanha T.M., Kubota L.T. (2022). DNA super-lattice-based aptasensor for highly sensitive and selective detection of cortisol. Biosens. Bioelectron. X.

[25] Pramanik H., Basu S. (2011). Cyclic Voltammetry of Oxygen Reduction Reaction Using Pt-based Electrocatalysts on a Nafion-bonded Carbon Electrode for Direct Ethanol Fuel Cell. Indian Chem. Eng..

[26] Adachi T., Nakamura Y. (2019). Aptamers: A Review of Their Chemical Properties and Modifications for Therapeutic Application. Molecules.

[27] Urmann K., Modrejewski J., Scheper T., Walter J.-G. (2017). Aptamer-modified nanomaterials: Principles and applications. BioNanoMaterials.

[28] Li M.J., Liu C.M., Bin Cao H., Zhang Y. (2013). Surface Charge Research of Graphene Oxide, Chemically Reduced Graphene Oxide and Thermally Exfoliated Graphene Oxide. Adv. Mater. Res..

[29] Deshmukh K., Basheer Ahamed M., Deshmukh R.R., Khadheer Pasha S.K., Bhagat P.R., Chidambaram K., Sadasivuni K.K., Ponnamma D., Kim J., Cabibihan J.J., AlMaadeed M.A. (2017). 3-Biopolymer Composites With High Dielectric Performance: Interface Engineering. Biopolymer Composites in Electronics.

[30] Ilango S., Vidjayacoumar B., Gambarotta S. (2010). Samarium complexes of a σ-/π-pyrrolide/arene based macrocyclic ligand. Dalton Trans..

[31] Hung H.M., Duc L.M., Dieu T.V., Trung V.Q. (2013). Molybdate Doped Polypyrrole: Preparation, Properties and Application. https://www.researchgate.net/profile/Vu-Quoc-Trung/publication/256199321_Molybdate_Doped_Polypyrrole_Preparation_Properties_and_Application/links/02e7e521ff49a598fc000000/Molybdate-Doped-Polypyrrole-Preparation-Properties-and-Application.pdf.

[32] Moghaddam A.Z., Bojdi M.K., Nakhaei A., Ganjali M.R., Alizadeh T., Faridbod F. (2018). Ytterbium tungstate nanoparticles as a novel sorbent for basic dyes from aqueous solutions. Res. Chem. Intermed..

[33] Settu K., Liu J.-T., Chen C.-J., Tsai J.-Z. (2017). Development of carbon−graphene-based aptamer biosensor for EN2 protein detection. Anal. Biochem..

[34] Majumder M., Choudhary R.B., Thakur A.K., Karbhal I. (2017). Impact of rare-earth metal oxide (Eu2O3) on the electrochemical properties of a polypyrrole/CuO polymeric composite for supercapacitor applications. RSC Adv..

[35] Jadhav N., Jensen M.B., Gelling V. (2015). Tungstate and vanadate-doped polypyrrole/aluminum flake composite coatings for the corrosion protection of aluminum 2024-T3. J. Coat. Technol. Res..

[36] Shahri Z., Salavati-Niasari M., Mir N., Kianpour G. (2014). Facile synthesis and characterization of nanostructured flower-like copper molybdate by the co-precipitation method. J. Cryst. Growth.

[37] Mani K.P., Vimal G., Biju P.R., Joseph C., Unnikrishnan N.V., Ittyachen M.A. (2015). Optical Nonlinearity and Photoluminescence Studies of Red Emitting Samarium Molybdate Nanophosphor. ECS J. Solid State Sci. Technol..

[38] Maheswari N., Muralidharan G. (2017). Controlled synthesis of nanostructured molybdenum oxide electrodes for high performance supercapacitor devices. Appl. Surf. Sci..

[39] Chithambararaj A., Sanjini N.S., Bose A.C., Velmathi S. (2013). Flower-like hierarchical h-MoO3: New findings of efficient visible light driven nano photocatalyst for methylene blue degradation. Catal. Sci. Technol..

[40] Deepa A.V., Priya M., Suresh S. (2016). Influence of Samarium Oxide ions on structural and optical properties of borate glasses. Sci. Res..

