# Supplementary material for: A Novel Electrochemical Aptasensor Based on a New Platform of Samarium Molybdate Flower-like Nanoparticles@Poly(pyrrole) for Non-Invasive Determination of Saliva CORTISOL
Source: Biosensors (Basel). 2022 Sep 3;12(9):720. doi: 10.3390/bios12090720 (PMC9496328; doi:10.3390/bios12090720)
Supplement: Supplementary file 1 [file biosensors-12-00720-s001.zip › biosensors-1871610-supplementary.pdf]

Supplementary

A Novel Electrochemical Aptasensor based on a New Platform of Samarium Molybdate Flower-like Nanoaprticles@Poly(pyrrole) for Non-invasive Determination of Saliva Cortisol

Zahra Rezapoor-Fashtali,[1] Mohammad Reza Ganjali [1,2,*] and Farnoush Faridbod[1]

[1] Center of Excellence in Electrochemistry, School of Chemistry, College of Science, University of Tehran, Tehran, Iran
[2] National Institute of Genetic Engineering and Biotechnology (NIGEB), Tehran, Iran
* Correspondence: ganjali@ut.ac.ir; Tel.: +98-2188356145

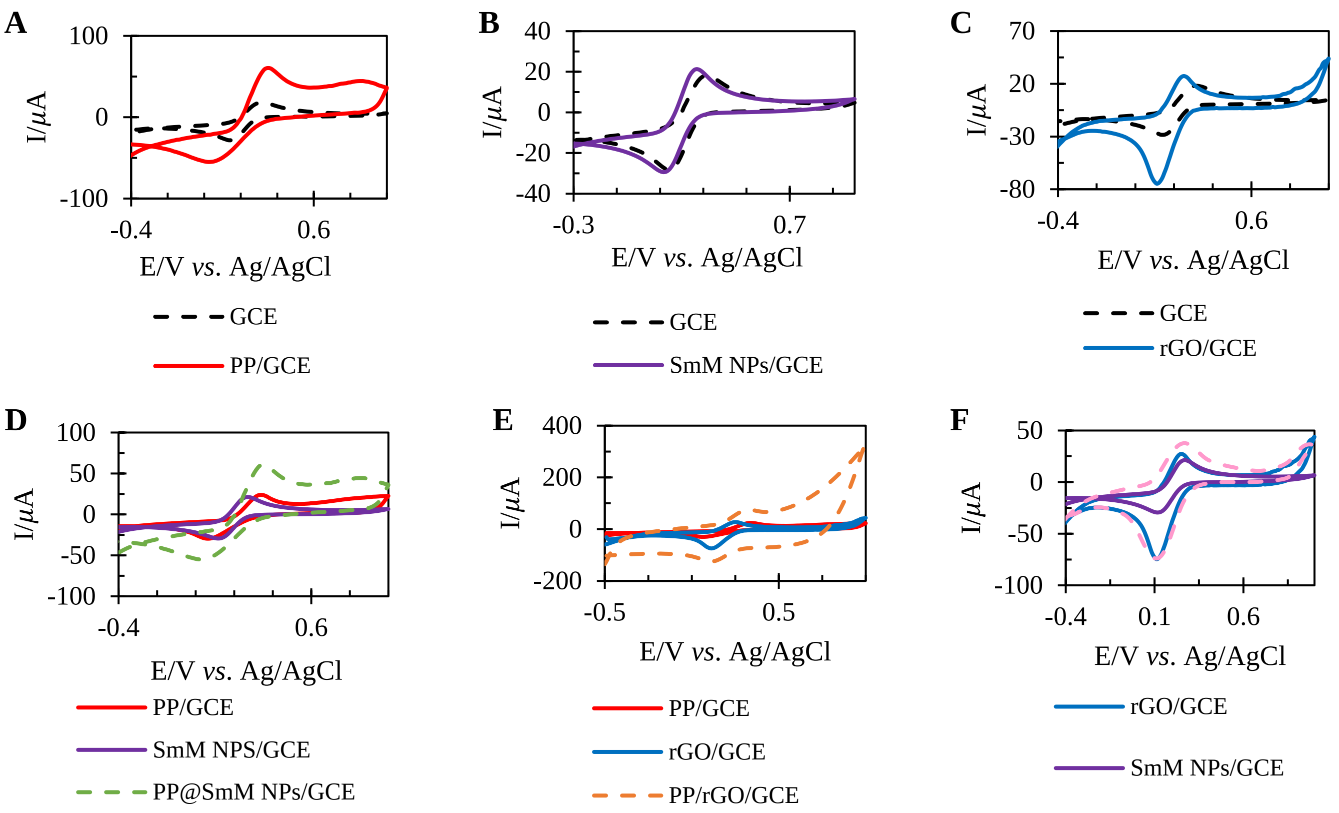

Figure S1. Comparison of electrode surface with the help of cyclic voltammetry *vs*. Ag/AgCl/KCl (3 mol/L) at potential scanning rate 0.1 V/s in ferricyanide 0.01 mol/L, PBS 0.01 mol/L, pH 7.4: A) the PP/GCE and the bare electrode; B) the SmM NPs/GCE and the bare electrode; C) the rGO/GCE and the bare electrode; D) the PP/GCE, SmM NPs /GCE with the SmM NPs@PP/GCE; E) the PP/GCE, rGO/GCE with the rGO/PP/GCE; F) the SmM NPs/GCE, rGO/GCE with the rGO/SmM NPs/ GCE

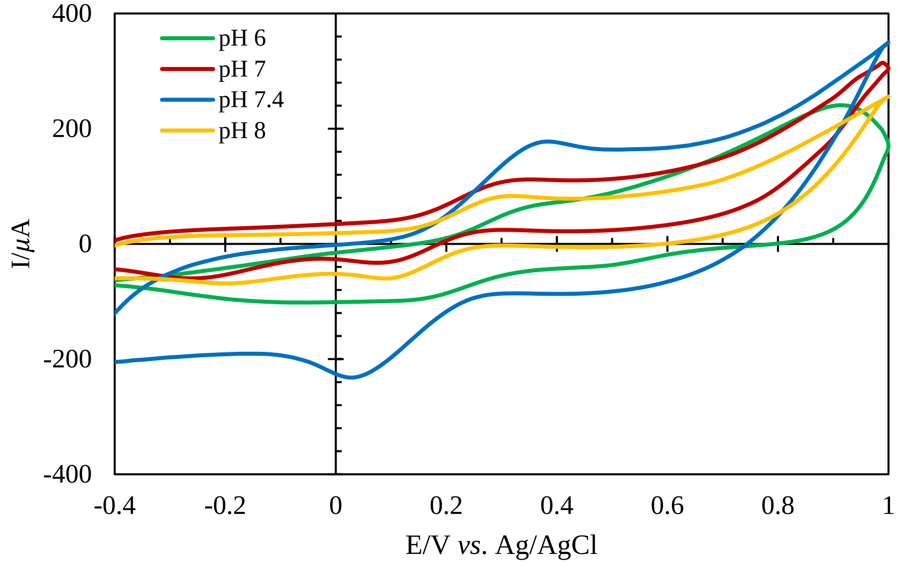

Figure S2. pH studies for the aptamer/rGO/SmM NPs@PP/GCE with the help of cyclic voltammetry *vs.* Ag/AgCl/KCl (3 mol/L) at potential scan rate 0.1 V/s in ferricyanide 0.01 mol/L, PBS 0.01 mol/L

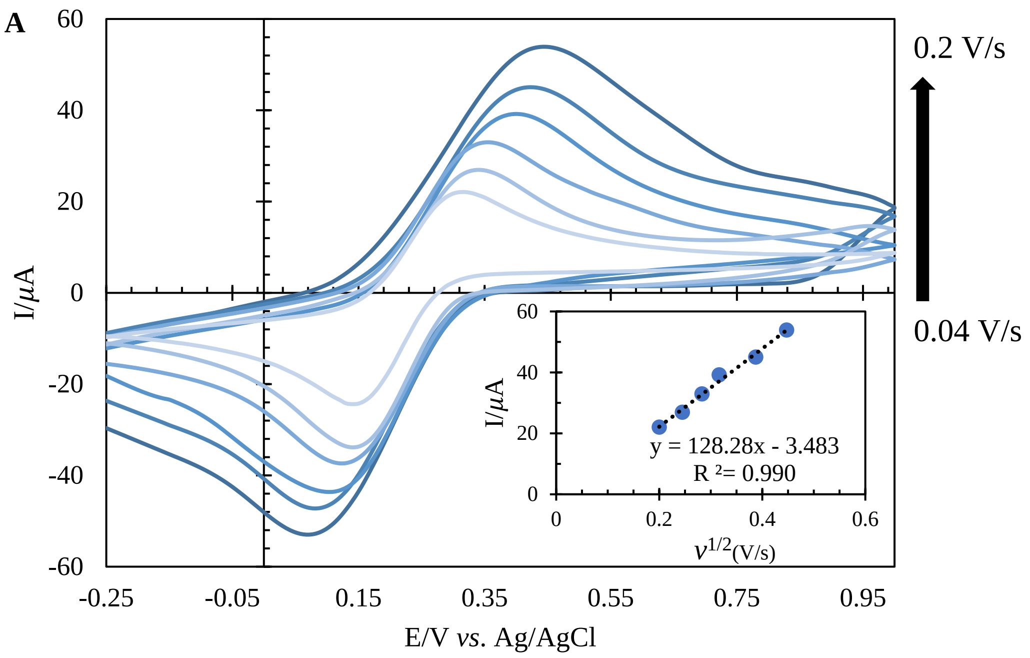

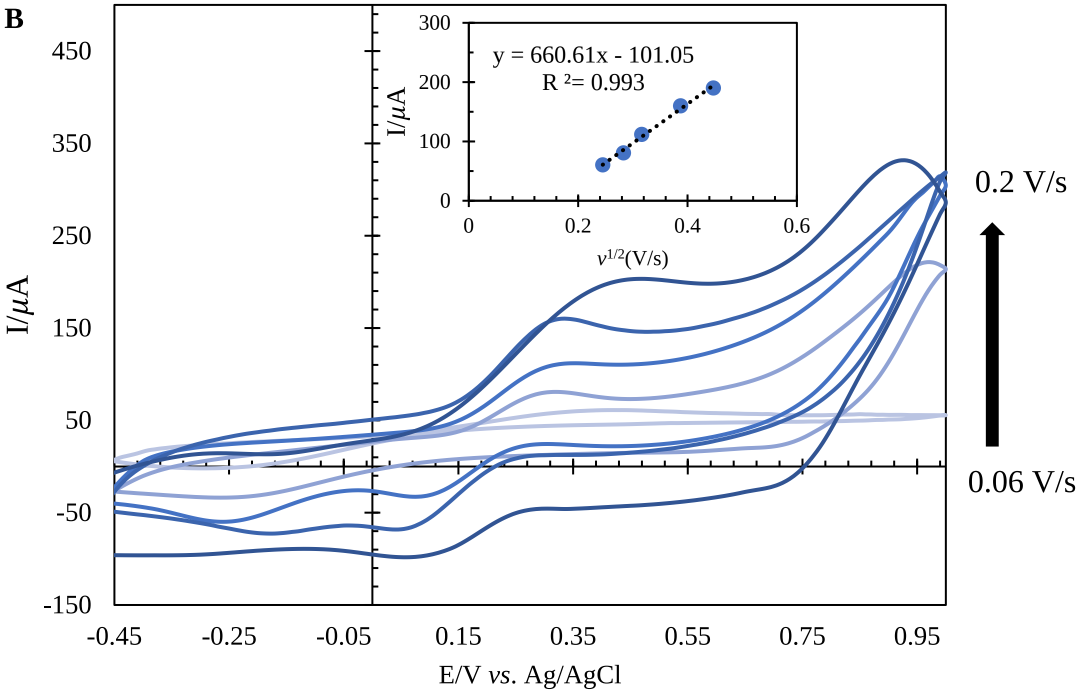

Figure S3. Investigation of the potential scanning rate on anodic currents of ferricyanide 0.01 mol/L, PBS 0.01 mol/L, pH 7.4 with the help of cyclic voltammetry *vs.* Ag/AgCl/KCl (3 mol/L) and the Randles-Sevcik equation to calculate the effective cross-section of (A) the bare GCE electrode; (B) the aptamer/rGO/SmM NPs@PP/GCE
